# Long-Term Variations in Habitat Use of Humpback Dolphins Due to Anthropogenic Activities in Western Pearl River Estuary

**DOI:** 10.3390/ani14233381

**Published:** 2024-11-24

**Authors:** Xinxing Wang, Min Li, Liang Fang, Tao Chen, Wenhua Liu

**Affiliations:** 1 South China Sea Fisheries Research Institute, Chinese Academy of Fishery Sciences, Guangzhou 510300, China; wangxinxing@scsfri.ac.cn (X.W.); limin@scsfri.ac.cn (M.L.); fangliang123_fl@163.com (L.F.); 2 Marine Biology Institute, Shantou University, Shantou 515063, China

**Keywords:** Indo-Pacific humpback dolphins, habitat use, core habitat, anthropogenic disturbance, western Pearl River Estuary, aquaculture

## Abstract

Marine mammals near coastlines are highly vulnerable to human activities like rapid industrialisation, port construction, and sea reclamation, which can alter their habitat use. This study examines changes in the habitat use of Indo-Pacific humpback dolphins in the western Pearl River Estuary (WPRE) by employing a kernel density estimation model that considers physical barriers. Sighting records from systematic surveys in 2007–2008 and 2019–2020, along with remote sensing data, were used to analyse changes in shorelines and areas affected by maritime projects since 1973. Approximately 552.98 km^2^ of water was permanently lost to reclamation between 1973 and 2020. In 2007–2008, dolphins preferred natural shorelines, while reclamation drove them away from artificial ones. By 2019–2020, their core habitat had decreased by two-thirds, with some areas disappearing, likely due to aquaculture expansion. These results highlight the importance of adopting improved environmental assessment methodologies in the planning and regulation of aquaculture activities in the WPRE to better protect the dolphin habitat.

## Simple Summary

Marine mammals living near coastlines are especially vulnerable to disturbances from anthropogenic activities. In the Pearl River Estuary, rapid industrialisation and urbanisation have led to significant habitat changes for Indo-Pacific humpback dolphins due to port construction and land reclamation. This study employed a new model to assess how the habitat use of these dolphins has evolved over time, comparing data from 2007–2008 and 2019–2020. By analysing satellite imagery and dolphin sighting data, the study revealed that over 550 km^2^ of water has been lost to development since 1973. The findings indicate that dolphins initially favoured natural shorelines but have been increasingly displaced by artificial ones. By 2019–2020, the core habitat area has contracted by approximately two-thirds compared to 2007–2008. Some key habitats have disappeared, likely due to aquaculture expansion. These results underscore the importance of adopting improved environmental assessment methodologies in development planning within the Pearl River Estuary to preserve the remaining dolphin habitat.

## 1. Introduction

Marine mammalian species inhabiting coastal areas, such as the Indo-Pacific humpback dolphin (*Sousa chinensis*, hereafter referred to as the humpback dolphin), are highly vulnerable to environmental problems caused by anthropogenic activities [1]. Humpback dolphins are small odontocetes widely distributed at depths shallower than 20 m along the coastal and estuarine waters of the Indian and Western Pacific Oceans [2,3,4].

The geographic range of humpback dolphins extends from central China—with the northernmost sighting recorded in the Yellow Sea in the east—southward throughout Southeast Asia and westward along the coastal rim of the Bay of Bengal, reaching as far as the Orissa coast in eastern India [5,6,7]. Humpback dolphins are primarily found in the southeast coastal waters of China: they have been detected in Xiamen Bay and the western coast of Taiwan [8,9,10], Shantou [11], the Pearl River Estuary to the Moyang River Estuary [3,12,13,14,15], the eastern waters of Zhanjiang [16], the northern Beibu Gulf [17,18,19], and the southwestern part of Hainan Island [20]. The humpback dolphin population in the Pearl River Estuary–Moyang River Estuary is the largest known population of this species, with an estimated abundance of more than 2600 individuals [12].

However, the distribution of humpback dolphins along coastal waters exposes them to the impacts of various human activities, posing a substantial threat to their populations [1]. In fact, humpback dolphins have been classified as a first-class national protected animal in China since 1988; in 2015, the International Union for the Conservation of Nature Red List of Threatened Species updated its conservation status from ‘near threatened’ to ‘vulnerable’ [21].

Anthropogenic habitat changes have altered the distribution patterns, habitat utilisation, and social structure of humpback dolphins. As dolphins select various habitats for activities, namely foraging, resting, and socialising [22], prolonged habitat loss adversely affects their living environment, leading to changes in their utilisation range and core habitat [15]. The present disjointed distribution of humpback dolphins off the west coast of Taiwan is likely attributed to varying degrees of habitat degradation rather than to the natural patchiness of the environment [23]. The offshore wind farm construction along Taiwan’s west coast has intensified threats to the critically endangered Taiwanese white dolphin, especially through increased vessel traffic and pile-driving noise that disrupt their habitat [24]. Wang et al. discovered a substantial shift in the distribution of humpback dolphins from inshore to offshore waters and away from artificial shorelines in Xiamen Bay, as well as a noteworthy shift in their core habitat from the original coastal areas to mid-channel waters in different years [25]. Additionally, anthropogenic habitat degradation may affect interactions among dolphin communities [26].

Over the past four decades, various irreversible human activities, including land reclamation, coastal development, and port construction, have been observed in the western Pearl River Estuary (WPRE). These activities not only alter the physical environment but also disrupt the underwater acoustic environment, impacting dolphin behaviour and habitat use [27,28,29,30]. The aquaculture areas within the WPRE have also expanded conspicuously. However, the potential impact of these anthropogenic activities on humpback dolphin populations remains poorly understood. Protecting humpback dolphins from habitat degradation due to anthropogenic factors requires that comprehensive and long-term survey data on dolphin distribution and habitat preferences in coastal waters occupied by vast offshore engineering areas be urgently obtained and analysed.

Hence, in this study, the responses of humpback dolphins to various anthropogenic stressors, including artificial coastlines and aquaculture, were analysed by utilising the sighting data collected by performing the systematic line transect method in two periods and referring to a series of Landsat images. Additionally, the effectiveness of existing protected areas and recommendations for habitat restoration in the context of conservation management was determined.

## 2. Materials and Methods

### 2.1. Study Area

**Figure 1:**
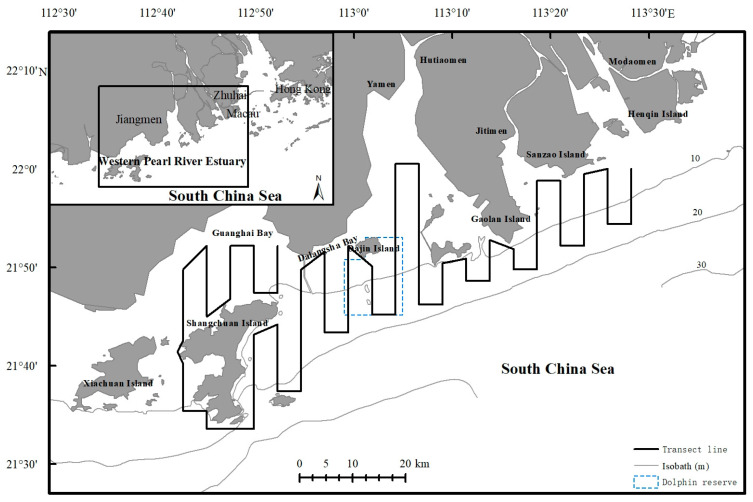
Study areas in western Pearl River Estuary demarcated with transect lines.

The Pearl River, which has a catchment area of 415,000 km^2^ [31], is the largest river in South China. Its delta, which spans an area of 50,000 km^2^, is second only to the Yangtze River delta within China. The river comprises eight distributaries that either directly or indirectly flow into the South China Sea. Four of these distributaries—Modaomen, Jitimen, Hutiaomen, and Yamen—discharge into the WPRE (situated between 21.45–22.23° N and 112.48–113.66° E), as illustrated in Figure 1. A series of parallel transect lines 3–4 km apart and generally perpendicular to major coastlines was established in the WPRE. As shown in Figure 1, the transect lines extended from the 3 m isobaths nearshore to the 20 m isobaths offshore. The total length of the transect line was 421 km, covering a survey area of approximately 1930 km^2^ in the WPRE.

### 2.2. Data Collection

The standard line transect method was employed for vessel surveys in the WPRE throughout the study period [3,32,33]. Between 2007 and 2020, 16 line transect surveys were conducted in the WPRE. These surveys were divided into two stages: the first stage involved 12 surveys, with 1 survey conducted each month from August 2007 to July 2008, comprising 6 surveys during the wet season and 6 during the dry season, and the second stage was 6 months (July 2019–April 2020). From 2019 until 2020, three surveys were conducted during each wet and dry season (July, August, October, November, and December in 2019 and April in 2020). Parallel transect lines were set up perpendicular to the major coastlines at approximately 3 km intervals. These lines were strategically designed to cover the survey area evenly and ensure that different sections of the WPRE were represented (Figure 1).

For the line transect vessel surveys, two shrimp trawlers with open upper decks were used for unobstructed visibility. The survey vessels maintained a constant speed of 13–15 km/h as they moved along different transect lines. Observations were from the flying-bridge area 4–5 m above sea level, under favourable weather conditions (Beaufort scale 0–5, no heavy rain, and visibility exceeding 1200 m). When a group of humpback dolphins was spotted, the course of the vessel was adjusted to match that of the group. Data on the position, distance travelled, and speed of the vessel were obtained using a handheld GPS device. However, only the positions of the sighted groups recorded under calm conditions (Beaufort scale 0–3) were factored into the kernel density estimation. Although individual photo-identification was conducted during both survey periods, the results from this approach were not included in the current study, as this paper does not focus on abundance estimation. Instead, these data will be considered in a future study focused on abundance comparison. An in-depth discussion of the survey procedures is in [13].

### 2.3. Landsat Imagery and Habitat Loss Measurements

A series of Landsat images of the study area (paths 122/131, row 45) were sourced and downloaded from the United States Geological Survey website (http://glovis.usgs.gov/, accessed on 15 January 2022). High-quality images with <20% and 0% cloud coverage for the entire image and over land areas, respectively, were selected for analysis (Table 1). Different band composites were processed for these Landsat images to enhance the contrast between water and land, as reported by [25,34,35]. To assess changes in coastal waters, we followed the process approach described by [15,25]. The earliest available Landsat images of the study area date back to 1973 (Landsat 1 MSS); the Landsat 1 MSS images were referred to in identifying areas of habitat loss where native aquatic habitats were once present.

### 2.4. Data Analysis

Utilisation distribution (UD) refers to a probability density function that details the relative spatial use by an animal within a defined area based on a sample of the animal’s location [36]. To estimate the UD of humpback dolphins, all the coordinates of dolphin school sightings were projected onto the UTM49N coordinate system. The kernel density estimate (KDE) for the data on dolphin group sightings adhered to the protocols outlined by [37], specifically for ‘estimating a home range in an environment with barriers to movement’. Using the KDE method, the overall utilisation range and core area were calculated at the 95% (95% KDE) and 50% (50% KDE) UD levels, respectively. The output cell size and bandwidth value are the two crucial user-defined parameters for the ‘kernel interpolation with barriers’ tool; the output cell size was set to 200 × 200 m to ensure that sufficient information from narrow study areas was incorporated [38]. The kernel function was designated as a first-order polynomial, and the ridge parameter maintained its default value of 50. The bandwidth value was determined by performing the least-squares cross-validation procedure [39].

The average proximity of the sighting location to the nearest artificial shoreline (APAS) and nearest natural shoreline (APNS) were calculated for the year of sampling. These proximity measures captured the relative distance of dolphin groups to shorelines and habitat loss sites during the two stages of the survey. A paired *t*-test was performed in R to compare the differences between the APAS and APNS in the same survey stage. Additionally, the paired comparison test for means was run in R to analyse variations in the APAS between the two stages. The results were considered statistically significant at *p* < 0.05.

The areas occupied by oyster aquaculture zones were extracted from satellite images and assessed [15]. The total habitat range area of humpback dolphins was determined to ascertain the spatial overlap between the aquaculture zones and the habitat range of humpback dolphins in the WPRE. To evaluate the effectiveness of the Nature Reserve at conserving the humpback dolphin population in the WPRE over different periods, the overlap of the 50% KDE within the Nature Reserve was calculated and compared for both stages of the survey.

## 3. Results

### 3.1. Summary of Survey Effort and Humpback Dolphin Sightings

A transect line of 4857 and 2063 km was established in the WPRE in 2007–2008 and in 2019–2020, respectively. Approximately 76.3% of the survey effort occurred under favourable sea conditions, characterised as a sea state ≤ 3, and accompanied by good visibility. A total of 155 and 109 groups of humpback dolphins were sighted in the WPRE in 2007–2008 and 2019–2020, respectively. Most of these sightings occurred during one-effort searches. To ensure accuracy and eliminate potential errors from adverse weather conditions, only the sighting data obtained under favourable sea conditions were analysed in this study.

### 3.2. Extent of Habitat Loss and Change in Sighting Location

**Figure 2:**
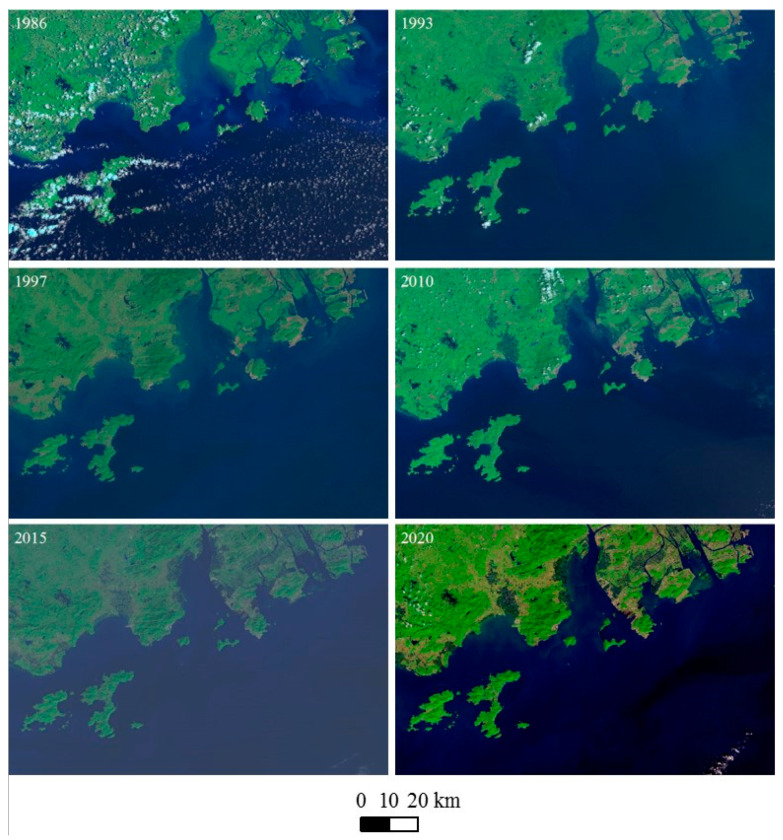
A series of Landsat images of western Pearl River Estuary, 1973–2020.

**Figure 3:**
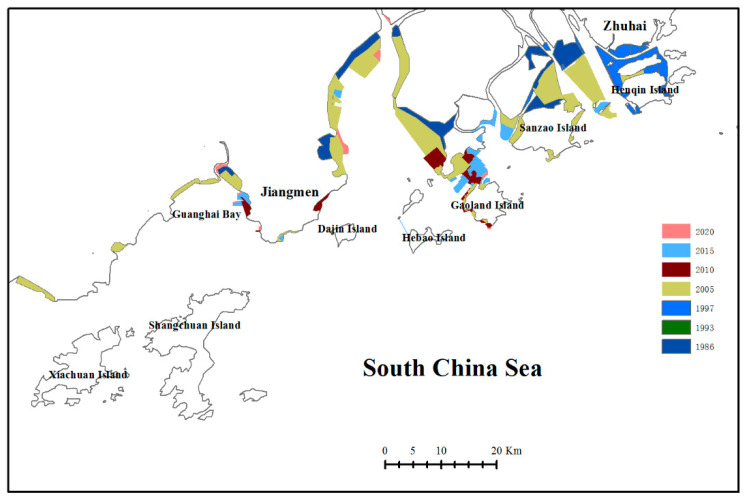
Sites where water was displaced by artificial landscapes (coastal constructions and land reclamation) in the western Pearl River Estuary.

**Figure 4:**
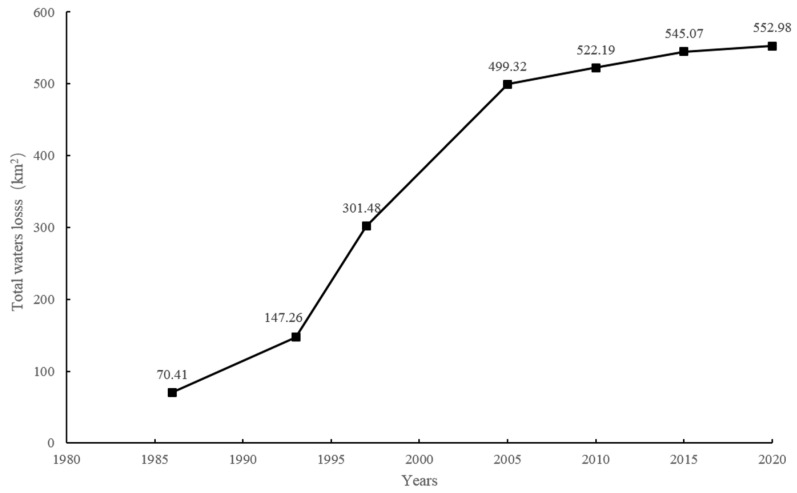
Area of total sea water surface loss from 1973 to 2020.

Coastal construction and land reclamation from the sea has altered the natural shoreline of the WPRE, resulting in the permanent loss of coastal sea water areas and volumes. The analysis of Landsat images captured from 1973 to 2020 (Figure 2) revealed substantial shoreline changes, highlighting the extent of habitat loss since 1973 (Figure 3). The total area of habitat loss amounted to 499.32 km^2^ from 1973 to 2005. However, the rate of habitat loss declined, with an area of 64.06 km^2^ lost between 2005 and 2020 (Figure 4).

**Figure 5:**
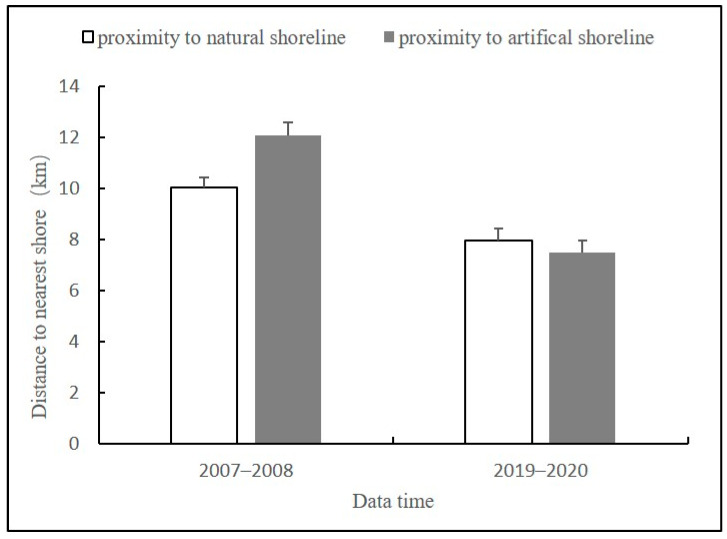
Proximity of humpback dolphins to the nearest natural shoreline and artificial shoreline, 2007–2020. The black vertical lines represent the standard error (SE) of the mean distances.

The locations where humpback dolphins are sighted indirectly indicate their habitat preferences, which are influenced by factors such as prey source availability and anthropogenic activities. The average proximity of dolphin group sighting locations to both artificial and natural shorelines during the two survey stages (Figure 5) was examined because it can temporarily or permanently alter the habitat preferences of dolphins. The differences between the APNS and APAS were highly significant (*p* < 0.001) in 2007–2008 and not statistically significant (*p* > 0.05) in 2019–2020. The difference in the APAS between the two stages was extremely significant (*p* < 0.001); similarly, the difference (*p* < 0.05) in the APNS between the two stages was significant.

### 3.3. Change in Humpback Dolphin Distribution and Core Habitat Utilisation

**Figure 6:**
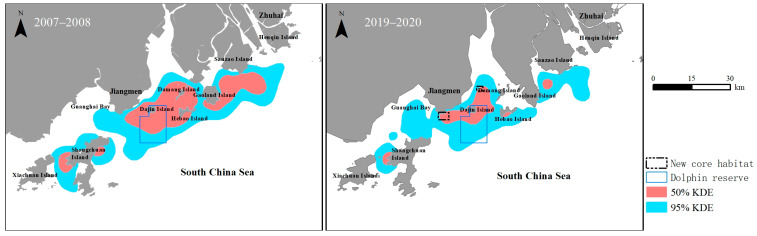
Extent of distribution and core habitat of humpback dolphins during the two stages of the survey period.

Humpback dolphins were observed throughout the study area during the survey period. The estimated distribution range (95% KDE) was 1289.45 km^2^ in 2007–2008 and 875.1 km^2^ in 2019–2020; additionally, the core habitat area (50% KDE) was 508.96 km^2^ in 2007–2008 and 157.7 km^2^ in 2019–2020 (Figure 6).

Regarding the longitudinal direction, the utilisation range of the dolphins did not change substantially between the two stages of the survey. However, the distribution pattern of the dolphins changed: the utilisation range was continuous in 2007–2008 but discrete in 2019–2020. The core habitat area of humpback dolphins in 2019–2020 was approximately two-thirds smaller than that in 2007–2008. The core habitat of humpback dolphins in the WPRE is mainly located around islands and near channels. In 2019–2020, some core habitats present in 2007–2008 disappeared, including the southeastern and southern waters of Sanzao Island, the western and eastern waters of Damang Island, and the northeastern waters of Shangchuan Island. Simultaneously, two new core habitats appeared in the northeastern waters of Dajin Island and the waters around Dalangsha Bay (Figure 6). The core habitat area within the dolphin reserve was approximately 54.28 km^2^ in 2019–2020, accounting for 34.42% of the total core habitat area (Table 2).

### 3.4. Analysis of Overlap Between Oyster Aquaculture Areas and Humpback Dolphin Habitats

**Figure 7:**
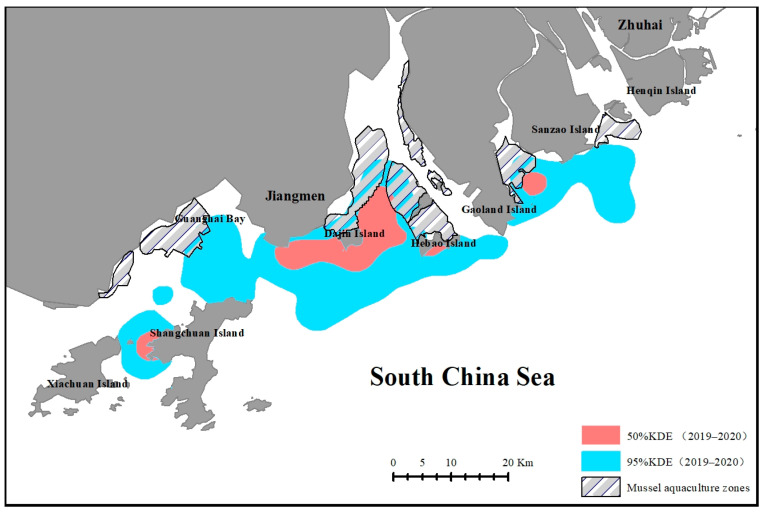
Polygon outlines of oyster aquaculture zones superimposed on the habitat range of humpback dolphins to evaluate overlap.

The analysis of the overlap between oyster aquaculture zones and humpback dolphin habitats revealed that some dolphin areas were encroached upon by the aquaculture zones. The main regions occupied by these oyster zones include the waters between Sanzao and Gaolin Islands, those between Hebao and Damang Islands, and the northwestern waters of Damang and Dajin Islands (Figure 7).

## 4. Discussion

This investigation is the first on habitat utilisation by the humpback dolphin population (the largest in the world) upon anthropogenic activities in the WPRE under various stressors. From 2007 to 2020, substantial changes have been observed in the distribution and habitat-use patterns of these dolphins. In 2007–2008, humpback dolphins were mainly located farther from artificial shorelines than from natural shorelines. This trend was reversed by 2019–2020, as the average distance of dolphin sightings from artificial shorelines was less than that from natural shorelines. Overall, when the two stages of the survey period were compared, the average distance of the dolphins from both shoreline types decreased. This finding indicates a substantial shift in the habitat-use pattern of humpback dolphins, evidenced by their general habitat becoming more restricted in 2019–2020 than in 2007–2008. As this shift brought their activities closer to the nearshore waters, their core habitat areas also diminished. The core habitats of humpback dolphins have vanished in regions such as the southeastern waters of Sanzao Island, western waters of Hebao Island, waters around Dali Island, and northeastern waters of Shangchuan Island. However, new core habitats have emerged northeast of Dajin Island and near Dalangsha Bay. Notably, because the overlap between the distribution of humpback dolphins and aquaculture zones affects the primary habitats of humpback dolphins in the WPRE, the findings of this study can inform conservation and management strategies for the humpback dolphins in this region and support the logical creation of Nature Reserves.

### 4.1. Distribution of Humpback Dolphins in Response to Shoreline Changes

Throughout the survey period, the average distance of dolphin sightings from both the natural and artificial coastlines along the WPRE decreased. In 2007–2008, the preference of humpback dolphins for natural coastlines may be attributed to the extensive reclamation projects in coastal waters at that time; to avoid the disturbance in nearshore habitats, the dolphins moved farther from artificial coastlines. Such a behaviour was also documented in dolphins in Lingding Bay [15]. Moreover, because waters near natural coastlines are less-altered marine habitats compared artificial coastlines, natural coastlines are preferable habitats for dolphins. By 2019–2020, reclamation activities slowed considerably, resulting in minimal changes in both natural and artificial coastlines.

Although Wang demonstrated that the distribution of humpback dolphins in Xiamen waters shifted from nearshore to offshore areas over time, away from artificial coastlines [25], our analysis differed slightly. The primary habitats in Xiamen consist largely of the dolphins’ core areas near the shore; in contrast, most humpback dolphin habitats in our study area are non-core habitats, suggesting that dolphins in our region may still have access to parts of their historical range and may gradually return to these areas if conditions improve.

The significant difference in the average distance of dolphin sightings from the natural coastline between 2007–2008 and 2019–2020 (F = 1.37, *p* < 0.05) suggests that the humpback dolphins in the WPRE were influenced by anthropogenic activities during both stages of the survey period. Concurrently, a reclamation project was built in the northwest waters of Gaolan Island, and a connection project was initiated between Hebao and Damang Islands. Disturbances from these offshore constructions might have prompted the dolphins to move further towards the shore. Moreover, the proximity of dolphin sightings to the artificial shoreline decreased significantly between the two stages of the survey period (F = 1.36, *p* < 0.005), indicating that from 2007 to 2020, the habitat preferences of humpback dolphins in the WPRE have shifted to adjust to anthropogenic disturbances in the marine environment.

### 4.2. Changes in Habitat Use of Humpback Dolphins

The utilisation range and core habitat of the humpback dolphins in the WPRE decreased by 2019–2020. The distribution within the utilisation range was more condensed in 2019–2020 than in 2007–2008. Additionally, the humpback dolphin habitat on the eastern side of Shangchuan Island disappeared.

The distribution pattern of the core habitats indicates that humpback dolphins predominantly inhabit estuaries and coastal waters surrounding islands. However, the core habitats of humpback dolphins became more spatially concentrated in 2019–2020, with several core habitats, such as those in the waters southeast of Sanzao Island, south of Gaolan Island, around Hebao Island, and around Damang Island, disappearing. Conversely, new core habitats have emerged, primarily in the waters east and northwest of Dajin Island. This shift reflects a decrease in the overall area of core habitats, rather than a change in dolphin density.

The loss of the core habitat of humpback dolphins might be attributed to both the Hebao Island–Damang Island connection project and oyster farming, as these factors altered the original hydrological and sedimentary environments. Moreover, the oyster farming around Damang Island directly encroached on dolphin habitats, forcing the dolphins to relocate westward from their original territories. Similarly, the emergence of new core habitats in northeastern and western parts of Dajin Island by 2019–2020 can be attributed to the forced relocation of humpback dolphins due to the encroachment of oyster farming in the east and the formation of a new conducive habitat in the port area northwest of Dajin Island.

The spatial distribution of the core habitats of humpback dolphins in the Pearl River Estuary exhibited a pattern of contraction towards waters near the main channel and uninhabited islands, as noted by [15]. This pattern reflects the preference of dolphins for port areas and channels in the WPRE. The migration of core habitats might be driven by alterations in the distribution density of their primary food sources—food fish [40]. Despite frequent ship movements, the Yangtze finless porpoise has been observed foraging near port areas, which are teeming with food resources; their choice to forage in such areas suggests an increased reliance on these food-rich environments [41]. Because the northwest channel of Dajin Island is >10 m deep and acts as a deep-water trench connecting to the open sea, it may serve as a migratory route for bottom fish moving from the open sea to the port. Owing to waters near the channel and port area of the WPRE being abundant in fish resources, the core habitats of humpback dolphins are concentrated in these regions. Additionally, the seabed topography and hydrology near islands promote abundant fish resources, including several reef fishes; thus, these regions are vital habitats for white dolphins [42].

### 4.3. Relationship Between Aquaculture and Humpback Dolphin Habitat Use

In the WPRE, the aquaculture zones for oysters overlap with the habitat of humpback dolphins. In 2007–2008, no evidence of oyster farming was found within the humpback dolphin habitat. However, based on satellite imagery and field investigations, the oyster-farming area had expanded to approximately 300.25 km^2^ by 2019–2020. These aquaculture zones were predominantly located in the southeastern and southwestern waters off Sanzao Island, the northwestern waters off Gaolan Island, the northern waters around Hebao Island, all around Dajin Island, and the northwestern region of Guanghai Bay (Figure 7). Notably, the aquaculture zones in the southwestern waters of Sanzao Island, northern waters of Hebao Island, and northern waters of Dajin Island overlapped with humpback dolphin habitats.

The expansion of aquaculture has encroached on both the general and core habitats of humpback dolphins, shifting habitat utilisation patterns; its most significant impact is evident around Hebao and Damang Islands, where the establishment of aquaculture farms has led to the loss of the core habitats of humpback dolphins.

Different aquaculture species and methodologies may have various effects on cetaceans. For instance, bottlenose dolphins (*Tursiops* sp.) less frequently used the area they originally occupied when an oyster farm was established and became operational in that area [43]. Similarly, dusky dolphins (*Lagenorhynchus obscurus*) use the areas around green-lipped mussel (*Perna canaliculus*) farms less frequently than those in other nearby regions in Marlborough Sounds, New Zealand [44]. Moreover, pressure from aquaculture may exacerbate existing anthropogenic strains, such as those arising from boat traffic, on dolphin populations [45]. However, areas dedicated to mussel production are frequented by common bottlenose dolphins, evidenced by an increase in bottlenose dolphin sightings in and around mussel farms [46]. These findings suggest that the interactions between the habitat of cetaceans and aquaculture zones are influenced by both the type of aquaculture practice and specific cetacean species. Notably, no new core habitats for humpback dolphins were identified within the aquaculture regions of Guanghai Bay, implying that these aquaculture areas do not attract humpback dolphins. The establishment of aquaculture may be associated with the contraction of dolphin habitat, although further research is needed to explore this potential relationship.

### 4.4. Functional Analysis of the Dolphin Reserve and Recommendations for Humpback Dolphin Habitat Protection

Marine Spatial Planning (MSP), as promoted by the UN and UNESCO, serves as a valuable management tool for coordinating human activities in marine spaces, reducing conflicts, and supporting habitat conservation efforts. Implementing MSP could benefit the Jiangmen Chinese White Dolphin Municipal Nature Reserve by balancing the sustainable use of marine resources with conservation goals. In China, Nature Reserves are subject to strict regulations designed to prioritise conservation. Industrial developments are strictly prohibited within the reserves.

The Jiangmen Municipal People’s Government of Guangdong Province approved the establishment of the Jiangmen Chinese White Dolphin Municipal Nature Reserve (hereafter dolphin reserve) in December 2003; the Guangdong Provincial People’s Government officially approved the upgrading of the Jiangmen Chinese White Dolphin Municipal Nature Reserve to the provincial level in January 2007. The habitats covered by the functional areas of Nature Reserves, especially the coverage of core habitats, indirectly reflect the role of Nature Reserves. The distribution pattern and area of the core habitat located in the Nature Reserve changed. The core habitat area of humpback dolphins in the dolphin reserve accounted for 16.36% of all core habitats at this stage, and the core area and buffer zone of the dolphin reserve were the core habitats in 2007–2008. Similarly, the proportion of dolphin core habitats in the dolphin reserve increased to 34.42%, and the core habitat and buffer areas of the reserve also increased.

The augmented area and proportion of the core habitats of humpback dolphins within the dolphin reserve may be attributed to intensified anthropogenic activities such as offshore island connection projects and oyster farming surrounding the reserve. These activities likely exerted pressure on the foraging and movement patterns of the dolphins, causing them to migrate to the dolphin reserve. The shifts in humpback dolphin distribution mentioned here are based on survey results commissioned by the Jiangmen Chinese White Dolphin Municipal Nature Reserve. However, these findings have not yet been formally published. Consequently, re-evaluating and adjusting the scope and functional zones of the reserve is necessary to improve the reserve to reflect current habitat changes. However, challenges arise because the majority of waters in the northern region of the reserve have been developed. Additionally, with the amended drainage plan of the Taishan Nuclear Power Plant [47], the space available for adjustment is scant. Notably, the distribution of humpback dolphin core habitats between Shangchuan and the Xiachuan Islands remained stable during the two stages of the survey period; thus, this area is a suitable candidate for a new Nature Reserve. For effective habitat restoration, aquaculture zones near Dajin Island and north of Hebao Island should be reconsidered, with an emphasis on fulfilling the habitat requirements of humpback dolphins.

Further research should not only focus on individual reserves but also on broadening the ecological conservation strategy for the entire Pearl River Estuary and adjacent waters. These considerations could involve the creation of a national park aimed at preserving humpback dolphin populations within the Pearl River Estuary and safeguarding the biodiversity of the broader estuary ecosystem.

## 5. Conclusions

This study demonstrates significant alterations in the habitat use of Indo-Pacific humpback dolphins in the WPRE due to increasing anthropogenic activities, particularly land reclamation and aquaculture expansion. Between 2007–2008 and 2019–2020, the dolphins’ core habitats diminished by two-thirds, driven by shoreline modifications and offshore constructions, which have severely disrupted natural habitats. The proximity of dolphins to artificial shorelines has increased over time, reflecting a notable shift in core habitat as reclamation projects and aquaculture development have rendered natural coastlines less accessible.

The overlap between aquaculture zones and dolphin habitats poses a serious challenge to the species’ conservation, impacting both habitat availability and the dolphins’ behaviour. While the Jiangmen Chinese White Dolphin Municipal Nature Reserve has played a role in protecting some core habitats, growing human activities in surrounding areas necessitate an expansion and re-evaluation of protected zones. To ensure the long-term survival of the humpback dolphin population in the WPRE, stricter regulation of aquaculture practices and the establishment of additional conservation areas, such as a national park, are critical. These measures should focus on habitat restoration and broader ecological conservation strategies that preserve the biodiversity of the estuary. Implementing Marine Spatial Planning (MSP) could play a crucial role in harmonising human activities and conservation efforts within the WPRE. By coordinating land reclamation, aquaculture, and other developmental activities, MSP can help minimise habitat conflicts and promote the sustainable use of marine resources. Incorporating MSP into conservation strategies, alongside stricter regulations and expanded protected zones, would further support the long-term preservation of humpback dolphin habitats and the broader estuarine ecosystem.

## Figures and Tables

**Table 1:** Landsat images used to delineate coastline changes in the western Pearl River Estuary.

Year	Satellites	Sensors	Path/Row	Day of Year
19731225	L1	MSS	131/45	359
19860730	L5	TM	122/45	211
19881124	L5	TM	122/45	329
19930903	L5	TM	122/45	246
19971101	L5	TM	122/45	305
20050123	L5	TM	122/45	23
20100918	L5	TM	122/45	261
20150103	L8	TM	122/45	3
20190319	L8	TM	122/45	78
20201202	L8	TM	122/45	337

**Table 2:** Habitat area and percentage of core habitat area distributed in dolphin reserve during the two stages of the survey period.

Survey Time	Core Habitat Area (km^2^)	Core Habitat Area Distributed in Dolphin Reserve (km^2^)	Percentage (%)
2007–2008	508.96	83.40	16.38
2019–2020	157.70	54.28	34.42

## Data Availability

The data presented in this study are available on request from the corresponding author without undue reservation.
